# Optimization of the Fluorescent Protein Expression Level Based on Pseudorabies Virus Bartha Strain for Neural Circuit Tracing

**DOI:** 10.3389/fnana.2019.00063

**Published:** 2019-06-21

**Authors:** Fan Jia, Pei Lv, Huan Miao, Xiangwei Shi, Hongjun Mei, Li Li, Xiaoqin Xu, Sijue Tao, Fuqiang Xu

**Affiliations:** ^1^State Key Laboratory of Magnetic Resonance and Atomic and Molecular Physics, Key Laboratory of Magnetic Resonance in Biological Systems, Wuhan Institute of Physics and Mathematics, Chinese Academy of Sciences, Wuhan, China; ^2^Brain Research Center, Wuhan Institute of Physics and Mathematics, Chinese Academy of Sciences, Wuhan, China; ^3^University of the Chinese Academy of Sciences, Beijing, China; ^4^Department of Orthopaedics, The Fifth Hospital of Wuhan, Wuhan, China; ^5^Center for Excellence in Brain Science and Intelligence Technology, Chinese Academy of Sciences, Shanghai, China

**Keywords:** PRV Bartha strain, retrograde trans-multisynaptic tracer, neural circuit, PRV531, PRV724

## Abstract

Mapping the neural circuits facilitates understanding the brain’s working mechanism. Pseudorabies virus (PRV; Bartha stain) as a tracer can infect neurons and retrogradely transport in neural circuits. To illuminate the network, tracers expressing reporter genes at a high level are needed. In this study, we optimized the expression level of reporter genes and constructed two new retrograde trans-multisynaptic tracers PRV531 and PRV724, which separately express more robust green and red fluorescent proteins than the existing retrograde tracers PRV152 and PRV614. PRV531 and PRV724 can be used for mapping the neural circuit of the central nervous system (CNS) and the peripheral nervous system (PNS). Overall, our work adds two valuable tracers to the toolbox for mapping neural circuits.

## Introduction

One of the core tasks of modern neuroscience is to map neural circuits of the central nervous system (CNS) and the peripheral nervous system (PNS). Neural circuits are composed of neural cells and their synapses, which process specific kinds of information to send orders or receive orders for executing related behaviors. To depict the neural network, several tracers have been developed, such as Vesicular stomatitis virus and Herpes simplex virus type 1 (strain 129) can map the output information of a target brain region, while Rabies virus and Pseudorabies virus (PRV; Bartha strain) have the ability to reveal the input neural circuit. Among these tracers, the PRV Bartha strain is the only retrograde trans-multisynaptic tool, and it is indispensable to determining the input neural circuit of the CNS and PNS (Collins et al., 1999; Zhang et al., 2003; Kc et al., 2006; Zhao, 2008; Kirby et al., 2010; Gonzalez-Joekes and Schreurs, 2012; Chen et al., 2013; Griffiths, 2015; Yao et al., 2018; Jin et al., 2019).

To facilitate visualizing the neural network, nonessential genes of virus can be replaced by the reporter genes expression cassette (Smith et al., 2000; Banfield et al., 2003; Wickersham et al., 2007; McGovern et al., 2012). The gG gene is nonessential for PRV replication, and a previous study engineered a retrograde trans-synaptic tracer by inserting a fragment of cytomegalovirus’s immediate early promoter, a single copy of reporter and a simian virus 40 poly(A) signal into the gG location of the PRV Bartha genome (Smith et al., 2000), which can be used for delineating the input neural circuit (Kondoh et al., 2016). In addition, enhancing the expression level of the reporter facilitates the observation of the labeled neural circuit. Therefore, to achieve this aim, we constructed a trans-multi-synaptic tracer based on the PRV Bartha strain by inserting the CAG promoter, β-globin intron, three copies of enhanced green fluorescent protein (EGFP; or three copies of mRuby3), woodchuck hepatitis virus post-transcriptional regulatory element (WPRE) and bovine growth hormone polyadenylation signal (BGHpA) into the gG location of the PRV Bartha genome (named as PRV531 and PRV724, respectively), which can express robust fluorescent protein and can be used for mapping the neural circuit. This new strategy can also be used for preparation of other sensitive tracers.

## Materials and Methods

### Preparation of Plasmids

In this study, nine plasmids were constructed to determine the optimized arrangement for preparing the new retrograde tracer based on PRV. To construct the plasmid PS496 [pcDNA3.1(+)-left arm-Ubc-EGFP-WPRE-bGHpA-right arm], first, the fragment of the Ubc promoter-EGFP-WPRE was inserted into the digested pcDNA3.1(+) with MluI and ApaI by using a ClonExpress MultiS One Step Cloning Kit (Vazyme Biotech); second, the right arm covers from nucleotides 59,647–61,646 in the PRV Bartha strain (Genbank no. JF797217), which was engineered into the digested pcDNA3.1(+)-Ubc-EGFP-WPRE-bGHpA with AscI and PshAI to prepare pcDNA3.1(+)-Ubc-EGFP-WPRE-bGHpA-right arm; and finally, the left arm (nucleotides 56,692–58,691) was inserted into the digested pcDNA3.1(+)-Ubc-EGFP-WPRE-bGHpA-right arm with PacI to prepare plasmid PS496.

To construct the plasmid PS506 [pcDNA3.1(+)-left arm-Ubc-3×EGFP-WPRE-bGHpA-right arm], the fragment EGFP-F2A-EGFP-T2A-EGFP was synthesized, which replaced the EGFP using the AsiSI and SwaI treated plasmid PS496. For generation of the plasmid PS515 [pcDNA3.1(+)-left arm-Ubc-6×EGFP-WPRE-bGHpA-right arm], the fragment EGFP-F2A-EGFP-T2A-EGFP was inserted into the AgeI- and SwaI-treated plasmid PS506. In addition, rabbit β-globin intron was inserted into the plasmid PS506 treated with AsiSI to prepare the plasmid PS529. The CAG was inserted into the plasmid PS529 treated with ClaI and AsiSI to prepare the plasmid PS531. To construct the plasmid PS724, the fragment mRuby3-F2A-mRuby3-T2A-mRuby3 was synthesized, which replaced the EGFP-F2A-EGFP-T2A-EGFP using the AsiSI- and AgeI-treated plasmid PS531.

### Preparation of Recombinant Virus PRV531 and PRV724

To generate PRV531 and PRV724, the 2 μg of plasmid was separately transfected into BHK21 cells. After 6 h post-transfection, the transfected BHK21 cells were infected by the PRV Bartha strain (moi = 1), then the virus sample was collected at 2 days post-infection. Furthermore, the PRV531 and PRV724 were separately purified by isolating the EGFP-positive and red-positive plaques for four rounds. Briefly, the expression cassette was inserted into the middle of the gG gene of the PRV Bartha genome.

### Stereotaxic Microinjection in Mice Brain

All procedures were approved by the Animal Care and Use Committees at the Wuhan Institute of Physics and Mathematics, the Chinese Academy of Sciences. Animal experiments were performed as referred in previous studies (Jia et al., 2016, 2017). Briefly, PRV152 (2.3 × 10^9^ PFU/ml, 300 nl) was stereotaxically microinjected into the ventral posteromedial nucleus of the thalamus (VPM) of the 8-week-old male C57BL/6 mice (20–25 g), and PRV152 (2.3 × 10^9^ PFU/ml, 100 nl), PRV531 (1.6 × 10^9^ PFU/ml, 100 nl), PRV614 (2.4 × 10^9^ PFU, 100 nl) and PRV724 (2.1 × 10^9^ PFU, 100 nl) were stereotaxically microinjected into the ventral hippocampal commissure (VHC) of the 8-week-old male C57BL/6 mice (20–25 g). Then mice were anesthetized with chloral hydrate (400 mg/Kg) and placed in a stereotaxic apparatus (RWD, 68030, 68025). After 2 days, mice were deeply anesthetized with an overdose of chloral hydrate and were transcardially perfused with 0.9% saline followed by 4% paraformaldehyde solution. The brains were removed and post-fixed overnight in 4% paraformaldehyde before being sectioned into 30 μm slices. Imaging was performed by using the TCS SP8 confocal microscope (Leica). Fixed slices were immunostained with mice primary antibody against the PRV gB (or EGFP) and amplified with a Cy3 (or FITC)-conjugated anti-mice secondary antibody (Jackson), and slices were stained with DAPI and imaged using the TCS SP8 confocal microscope (Leica).

### Stereotaxic Microinjection in Rats’ Hindlimb Muscle

Eight-week-old male Sprague-Dawley rats (200–250 g) were anesthetized with chloral hydrate, then PRV531 (1.2 × 10^10^ PFU, 2 μl) was microinjected into the muscle of the hindlimb. After 6 days, rats were deeply anesthetized with an overdose of chloral hydrate and were transcardially perfused with 0.9% saline followed by 4% paraformaldehyde solution. The brain and spinal cord were removed and post-fixed overnight in 4% paraformaldehyde before being sectioned into 30 μm slices. Imaging was performed by using the TCS SP8 confocal microscope (Leica).

### Plaque Assay

Plaque assay was performed to determine the viral titer as referred to in a previous study (Jia et al., 2016). Briefly, each dilution (100 μl) sample was seeded to individual wells of 6-well plates containing BHK-21 cells and incubated under 5% CO_2_ at 37°C for 1 h, then the cells were overlaid with the first layer of agar. A second layer of agar containing neutral red was added at an indicated time point. Plaque numbers were counted after an additional 24 h of incubation.

### Western Blot

BHK-21 cell monolayers were cultured in a 3.5 cm dish and infected with PRV 531 (moi = 1). Then cells were collected and lysed at 48 h post infection, the cell lysate was analyzed on a 12% SDS-PAGE and then electro-transferred to PVDF Immobilon-P membranes (Millipore) blocked with 5% skim milk in TBST, which was then treated with PcAb against EGFP at a dilution of 1:7,000 for 1 h at room temperature. After washing three times in TBST, the secondary anti-mouse antibody conjugated to Horseradish peroxidase at a dilution of 1:10,000 was applied to the blots for 1 h at room temperature. To quantify the expression amount of EGFP, the β-actin was analyzed by using first antibody (1:5,000) and second antibody (1:10,000). Signal was detected chemiluminescently with ECL Western blotting reagent.

## Results

### PRV152 Cannot Express Robust Fluorescent Protein for Mapping Neural Circuit

**Figure 1:**
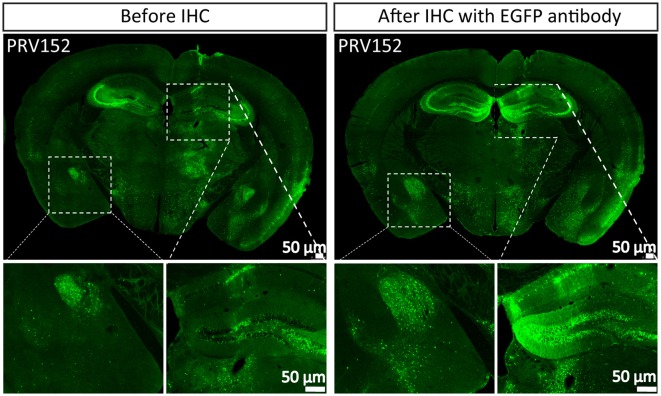
PRV152 has low enhanced green fluorescent protein (EGFP) expression level. PRV152 was injected into the ventral posteromedial (VPM) region of mouse brain. At 48 hpi, the mice were sacrificed, the brain slices were prepared and the EGFP signal was detected. The brain section was treated with EGFP antibody, and the resulting stronger EGFP signal was imaged.

Tracer is an important tool for mapping neural circuits. PRV152 as a trans-multisynaptic tool has been constructed by inserting the EGFP expression cassette into gG from the PRV Bartha strain (Smith et al., 2000), which has been widely used in depicting the neural network of the CNS and PNS (Collins et al., 1999; Zhang et al., 2003; Kc et al., 2006; Kirby et al., 2010; Gonzalez-Joekes and Schreurs, 2012; Chen et al., 2013; Yao et al., 2018; Jin et al., 2019). To highlight the labeled neurons, the more the EGFP amount, the better the information. PRV152 was stereotaxically microinjected into the VPM region of the mouse brain. The brain sections were prepared at 2 dpi, and the EGFP signals were imaged (Figure 1). The brain section was then treated with EGFP antibody and more EGFP neurons were detected (Figure 1), which indicates that PRV152 has the drawback of low EGFP expression level.

### Optimization of the Expression Level of Fluorescent Protein

**Figure 2:**
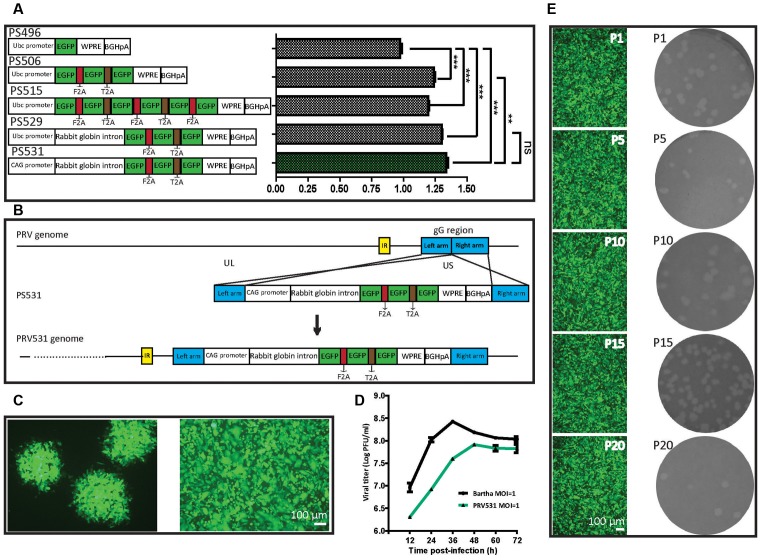
Optimization of EGFP expression level and generation of recombinant PRV531. **(A)** Comparison of expression levels of EGFP from different construction strategies. The expression plasmids were separately constructed based on the schematic design, then equal amounts of them were separately transfected into BHK21 cells. Student’s *t*-test was conducted to compare the difference of EGFP signals of plasmids PS496, PS506, PS515, PS529 and PS531. *N* = 3, “***” represents *p* < 0.001, “**” represents *p* < 0.01, “ns” represents *p* > 0.05. Ubc promoter, Ubiquitin C promoter; EGFP, Enhanced green fluorescent protein; F2A, Foot-and-mouth disease virus 2A; T2A, Thoseaasigna virus 2A; WPRE, Woodchuck hepatitis virus post-transcriptional regulatory element; BGHpA, Bovine growth hormone polyadenylation signal; CAG promoter, Actin promoter coupled with CMV early enhancer. **(B)** Cloning diagram of recombinant PRV531. The top represents PRV genome. The middle represents the expression cassette of plasmid PS531, which is flanked by the left homologous arm (left arm) and right homologous arm (right arm). The bottom represents the genome of recombinant PRV531. **(C)** Purification of recombinant PRV531. The plaque assay was performed on BHK21 cells. The single green plaque was picked and loaded into BHK21 cells. The purified PRV531 infects BHK21 cells. **(D)** The growth curve of PRV531 and its parent virus. Virus infects BHK21 at moi = 1 and the sample was collected at indicated time points (12, 24, 36, 48, 60 and 72 hpi). **(E)** Analysis of PRV531 stabilization. The virus was passaged on BHK21 cells for 20 rounds. Then the P1, P5, P10, P15 and P20 samples were selected to perform the plaque assay on BHK21 cells. These selected samples separately infect BHK21 cells and EGFP signals were imaged using a fluorescence microscope (IX73, Olympus).

To try to elevate the expression level of EGFP, we compared the expression efficiencies of one, three and six copies of EGFP and found that three EGFP copies can produce the brightest signal among them (Figure 2A). However, attempting to further increase EGFP expression level by adding its copy was inefficient. Previous studies show that intron combination with promoter can enhance gene expression level by augmenting mRNA synthesis through regulating the efficiency of transcription initiation and RNA polymerase II processivity (Fong and Zhou, 2001; Furger et al., 2002; Kwek et al., 2002; Montiel-Equihua et al., 2012; Du et al., 2014). Therefore, the rabbit β-Globin Intron II was added into the downstream of promoter, the intron indeed enhanced the EGFP expression level (Figure 2A). In addition, several reports show that the activity of CAG promoter is stronger than a subset of promoters (Qin et al., 2010; Chen et al., 2011). The CAG promoter replaced the Ubc promoter, which increased the expression level of EGFP (Figure 2A). These results indicate that an optimized version of EGFP expression cassette (PS531: CAG promoter- rabbit β-Globin Intron-3×EGFP-WPRE-BGHpA) was successfully constructed. Furthermore, using a similar strategy, PS724 was prepared by inserting the fragment mRuby3-F2A-mRuby3-T2A-mRuby3 into the plasmid PS531 treated with the AsiSI and AgeI.

### Preparation and Characterization of Recombinant PRV531 and PRV724

Next, the optimized EGFP expression cassette was inserted into the gG location of PRV Bartha by homologous recombination (Figure 2B). The purified recombinant PRV531 produced fluorescent plaque visualized using fluorescent microscopy and formed cytopathic plaque on BHK21 cells (Figure 2C). The PRV531 infected BHK21 cells and expressed EGFP (Figure 2C). In addition, the growth curve of the virus was determined by testing the viral titers of samples at indicated time points. We found that the amount of PRV531 increased from 12 hpi (hours post-infection), with a peak value around 10^8^ PFU/ml at 48 hpi (Figure 2D). Then the PRV724 was prepared using the similar strategy (Figure 6A).

**Figure 3:**
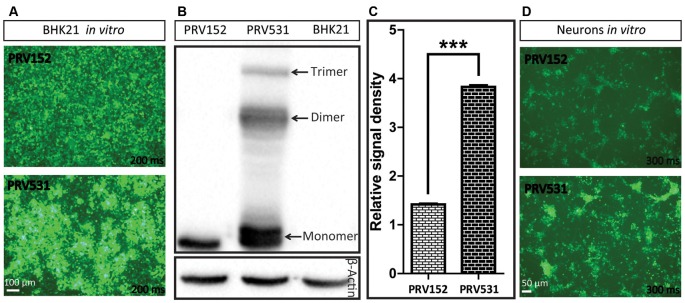
Comparison of EGFP expression level of PRV531 and PRV152 *in vitro*. **(A)** PRV531 and PRV152 separately infect BHK21 cells *in vitro* and the EGFP signals were detected using the same imaging parameter at 24 hpi. PRV531 expresses more EGFP than PRV152 *in vitro*. **(B,C)** The cell lysis of BHK21 cells infected with PRV531 and PRV152 at 48 hpi were collected and analyzed using EGFP antibody. Student’s *t*-test was conducted to compare the difference of EGFP expression levels of PRV531 and PRV152. *N* = 3, “***” represents *p* < 0.001. **(D)** PRV531 and PRV152 separately infect neurons *in vitro* and the EGFP signals were detected using the same imaging parameter at 24 hpi.

**Figure 4:**
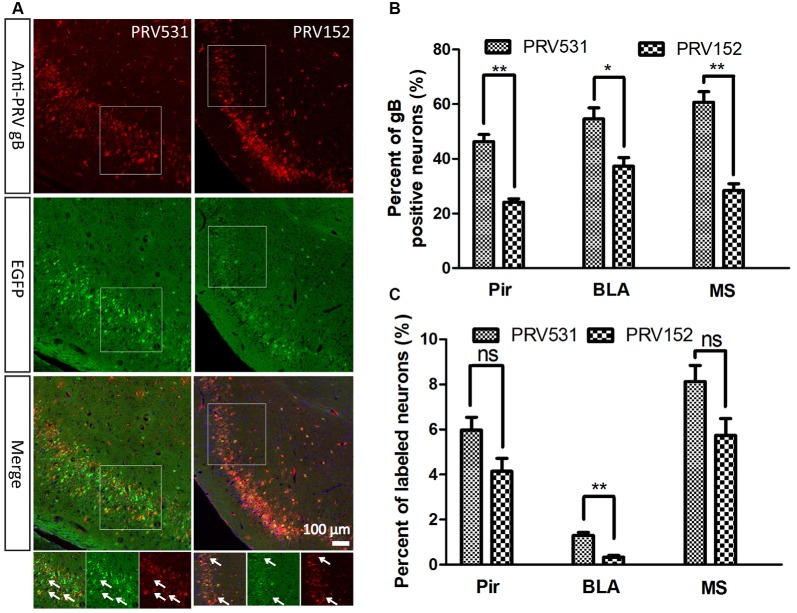
Comparison of EGFP expression level of PRV531 and PRV152 *in vivo*. **(A)** PRV531 (1.6 × 10^9^ PFU/ml, 100 nl) and PRV152 (2.3 × 10^9^ PFU/ml, 100 nl) were separately injected into the ventral hippocampal commissure (VHC) region of the mouse brains. At 48 hpi, the mice were sacrificed and the brain slices were prepared. The brain sections were treated with PRV-gB antibody, and more red signal was observed. **(B)** Three brain regions, Pir, MS, and BLA, were randomly selected for displaying the co-labeling efficiency of EGFP signals with gB positive neurons. Student’s *t*-test was performed. *N* = 3, “*” represents *p* < 0.05, “**” represents *p* < 0.01. **(C)** Furthermore, the EGFP expression level of PRV531 and PRV152 were analyzed *in vivo*. Three brain regions, Pir, MS and BLA, were randomly selected for analyzing the EGFP expression level of PRV 531 and PRV152. Student’s *t*-test was conducted. *N* = 3, “**” represents *p* < 0.01, “ns” represents *p* > 0.05. Pir, piriform cortex; MS, medial septal nucleus; BLA, basolateral amygdaloid nucleus, anterior part.

**Figure 5:**
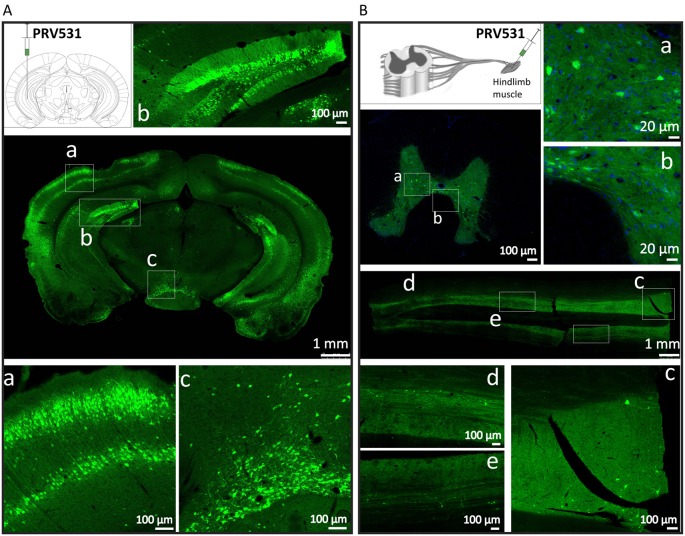
PRV531 labels the neural circuit of central nervous system (CNS) and peripheral nervous system (PNS). **(A)** Hundred nanoliter of PRV531 (1.6 × 10^9^ PFU/ml) was injected into the VHC region of mice brain. At 48 hpi, the mice were sacrificed and the brain slices were prepared. **(B)** The 2 μl of PRV531 (1.2 × 10^10^ PFU/ml) was injected into the hindlimb muscle of rat. After 6 dpi, EGFP positive signals were found in neurons located in spinal cord.

**Figure 6:**
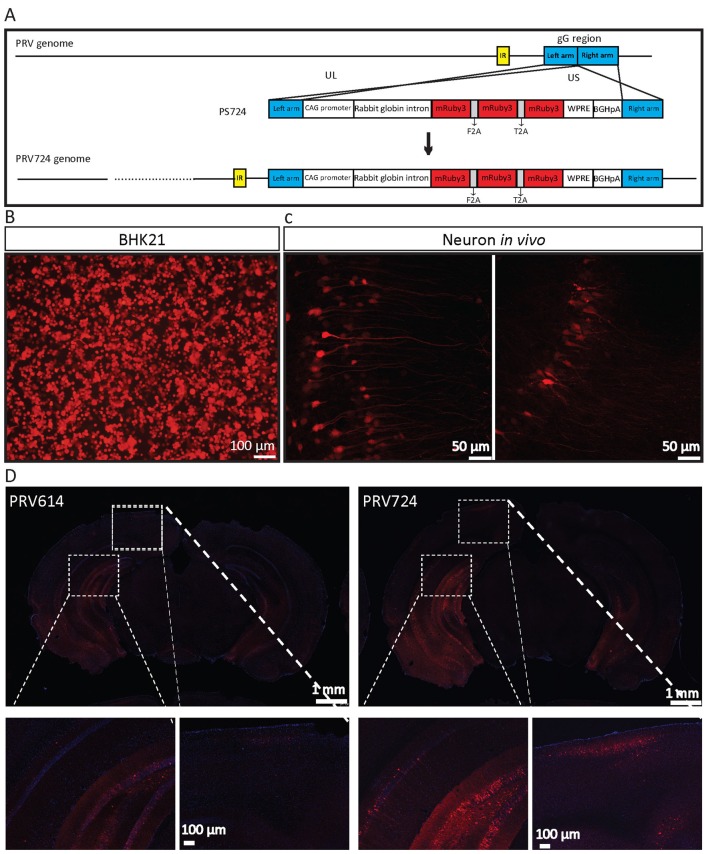
PRV724 was constructed and applied in mapping neural circuits. **(A)** Cloning diagram of recombinant PRV724. The top represents the PRV genome. The middle represents the expression cassette of plasmid PS724, which is flanked by the left homologous arm (left arm) and right homologous arm (right arm). The bottom represents the genome of recombinant PRV724. **(B)** The purified PRV724 infects BHK21 cells. **(C)** PRV724 labels the neural circuit. Then the 100 nl of PRV724 (2.1 × 10^9^ PFU/ml) was injected into the VHC region of the mouse brains and the red signals were imaged at 24 hpi. **(D)** PRV724 (2.1 × 10^9^ PFU, 100 nl) and PRV614 (2.4 × 10^9^ PFU, 100 nl) were separately injected into the VHC region of the mouse brains. At 48 hpi, the mice were sacrificed and the brain slices were prepared. The red signals were detected using the same imaging parameter between PRV724 and PRV614.

Reporter virus stably expressing reporter protein is important for its application in labeling neural circuits. Therefore, to detect the stability of PRV531, the virus was passaged on BHK21 cells for 20 rounds. The P1, P5, P10, P15 and P20 viruses produced similarly sized plaque and expressed EGFP on BHK21 cells (Figure 2E). Taken together, these results indicate that PRV531 and PRV724 were prepared successfully.

### PRV531 Expresses More EGFP Than PRV152 *in vitro* and *in vivo*

Next, the EGFP expression level of PRV152 and PRV531 was analyzed by fluorescent signal and western blot. We found that PRV531 produced more fluorescent signal than PRV152 in BHK21 cells and neurons (Figures 3A,D). The western blot showed that the EGFP amount of PRV531 expression is more than PRV152 (Figures 3B,C), and PRV531 produced monomer, dimer and trimer EGFP due to the insufficient cleavage of 2A (Figure 3B). These results indicate that the strategy for enhancing protein expression in the PRV Bartha strain is successful *in vitro*.

Furthermore, the EGFP expression level of PRV531and PRV152 was analyzed *in vivo*. A total of 100 nl of PRV152 (2.3 × 10^9^ PFU/ml) and PRV531 (1.6 × 10^9^ PFU/ml) was separately injected into the VHC region of the mouse brains. After 2 dpi (days post-infection), EGFP-positive brain regions were observed and the brain sections were treated with antibody against PRV-gB. It was found that the EGFP signals were not fully matched with the red signals (gB antibody; Figure 4A). The EGFP signal of PRV531 has a higher co-labeling ratio with gB-positive neurons than PRV152 (Figure 4B), which indicates that PRV531 expresses more EGFP than PRV152 *in vivo*. However, PRV531 and PRV152 have similar tracing patterns, such as Pir, BLA, and MS (Figure 4C). Collectively, these results show that the PRV531 expresses more EGFP than PRV152 *in vitro* and *in vivo*.

### PRV531 Labels Neural Network in CNS and PNS

Then, we tested the ability of PRV531 labeling neurons and spreading in neural circuits when it was separately injected into mouse brain and muscle. For the former 100 nl of PRV531 (1.6 × 10^9^ PFU/ml) was injected into the VHC region of mice brain and, after 2 dpi (days post-infection), EGFP-positive brain regions were observed (Figure 5A). Regarding the latter, 2 μl of PRV531 (1.2 × 10^10^ PFU/ml) was injected into the hindlimb muscle of rat and, after 6 dpi, EGFP-positive signals were found in neurons located in the spinal cord (Figure 5B). These results indicate that PRV531 can be used for mapping neural circuits in the CNS and PNS.

### PRV724 Expresses Robust Red Fluorescent Protein

Based on the above data, PRV724 expressing robust red fluorescent protein was generated using a similar strategy (Figure 6A). However, mRuby3 (improved brightness and photostability) was selected as the targeted red reporter gene (Bajar et al., 2016). PRV724 was prepared in BHK21 cells (Figure 6B). Then, the 100 nl of PRV614 (2.4 × 10^9^ PFU) and PRV724 (2.1 × 10^9^ PFU/ml) was injected into the VHC region of the mouse brain. After 2 dpi, mRuby3-positive brain regions were observed and the obvious neural fibers were detected (Figure 6C). Furthermore, PRV724 expresses more red fluorescent proteins than PRV152 (Figure 6D). These results indicate that PRV724 can express robust red fluorescent protein and be used for mapping neural circuits.

## Discussion

High expression level of reporter gene, such as EGFP, is important to visualize the structure network. PRV as a DNA virus vector has lower expression efficiency of heterologous gene than Rabies virus, Vesicular stomatitis virus, Semliki Forest virus (Beier et al., 2013; Haberl et al., 2015; Jia et al., 2019). Therefore, to elevate the expression level, we make an effort on the modulation of reporter gene copies, RNA transcription, post-transcriptional modification of RNA, RNA stability and protein translation.

To increase the expression level of genes, the direct method is to amplify the copy number of the inserted gene. Interestingly, we found that three copies of EGFP can produce more robust signal than one and six copies of EGFP (Figure 2A), which indicates that further increasing EGFP by adding more gene copies might be inefficient. Figure 3B shows that PRV531 expresses three main bands and lots of small bands were detected that may correspond to degradation fragments (Figure 3B). We speculated that polymers of EGFP are easily degraded, and the EGFP of six copies has a lower expression level than three copies due to degradation of EGFP. In addition, previous reports show that intron can significantly enhance the transcriptional products of genes to elevate the expression level protein (Le Hir et al., 2003; Nott et al., 2003; Moabbi et al., 2012). Based on these reports, we engineered the intron into the location between promoter and EGFP and found that the EGFP expression level is higher than intronless (Figure 2A). Furthermore, constitutive promoters, such as CMV, UBC and CAG, can be used to drive heterologous gene transcription to prepare RNA for protein expression. The strengths of these promoters usually are different in the same cellular contexts (Chen et al., 2011). Previous studies show that the CAG promoter is a strong promoter which has the ability to drive higher levels of protein expression in several cell lines compared to CMV and Ubc (Niwa et al., 1991; Chen et al., 2011). Indeed, we found that CAG promoter is stronger than Ubc promoter (Figure 2A). The WPRE can modulate RNA in post-transcriptional effect and is broadly used for enhancing gene expression in RNA and DNA viral vectors (Loeb et al., 1999; Zufferey et al., 1999). Therefore, the WPRE was placed between EGFP and the polyadenylation signal. Based on the optimized expression strategy, PRV531 and PRV724 were respectively prepared based on the PRV Bartha strain, which can produce robust green and red signals and be used in depicting the neural circuit (Figures 5, 6). Collectively, this work adds two new tools to the approaches for retrograde labeling neural circuits in the CNS and PNS.

## Data Availability

All datasets generated for this study are included in the manuscript.

## Ethics Statement

All procedures used were approved by the Animal Care and Use Committees at the Wuhan Institute of Physics and Mathematics, Chinese Academy of Sciences. All the experiments with viruses were performed in Biosafety Level 2 laboratory and animal facilities.

## Author Contributions

FJ and FX conceived the project and analyzed the data. FJ designed the experiments and wrote the manuscript. PL, HM, XS, HJM, LL, XX, FJ, and ST performed experiments.

## Conflict of Interest Statement

The authors declare that the research was conducted in the absence of any commercial or financial relationships that could be construed as a potential conflict of interest.
